# Skin nodules of distal-type epithelioid sarcoma^⋆^

**DOI:** 10.1016/j.abd.2021.08.014

**Published:** 2023-02-20

**Authors:** Vivian Spanemberg Macuglia, Juliano Peruzzo, Ariane Bastos Geller, Renan Rangel Bonamigo

**Affiliations:** a Department of Dermatology, Hospital de Clínicas de Porto Alegre, Porto Alegre, RS, Brazil; a Department of Dermatology, Hospital de Clínicas de Porto Alegre, Porto Alegre, RS, Brazil; b Faculty of Medicine ‒ Universidade Federal do Rio Grande do Sul, Porto Alegre, RS, Brazil

Dear Editor,

We describe a case of a 43-year-old man who was referred to the Dermatology Service, reporting the progressive appearance of painful suppurative nodules in the right lower limb for six months. The lesions started after penetrating trauma to the foot. He had hardened nodules, some of them ulcerated, on the lateral aspect of the right lower limb, in an ascending distribution (Fig. 1). Histopathology showed a malignant neoplasm infiltrating the skin and subcutaneous tissue, epithelioid cells with granulomatous morphology and an area of necrosis (Fig. 2). Cultures for bacteria, fungi, and mycobacteria showed no growth. Immunohistochemistry showed reactivity for INI-1 and lysozyme. There was focal reaction with CD68 and no reaction for EMA, CD34, desmin and S100, which is compatible with epithelioid sarcoma (ES). Staging showed ipsilateral inguinal lymph node enlargement measuring 3.1×2.0 cm. He underwent chemotherapy with doxorubicin with control of the appearance of new lesions and healing of old lesions.

**Figure 1:**
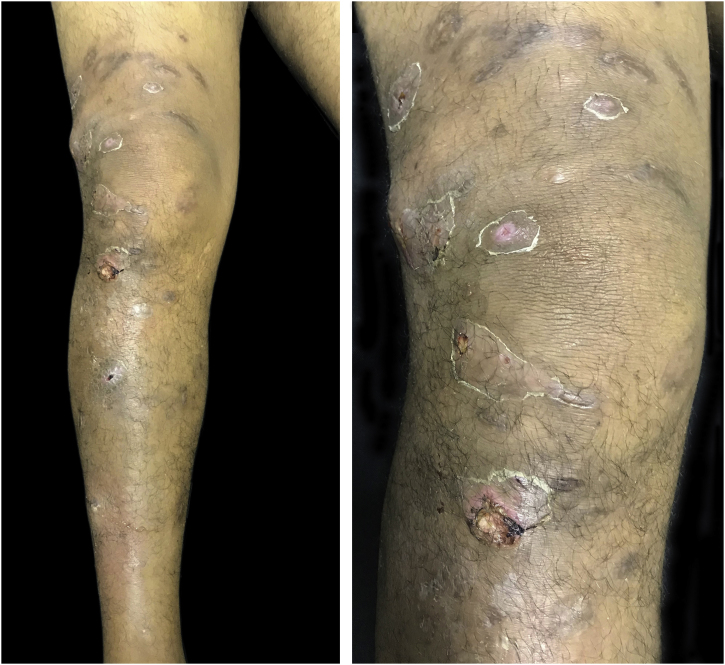
Erythematous-violaceous nodules, some of them ulcerated, in the right lower limb.

**Figure 2:**
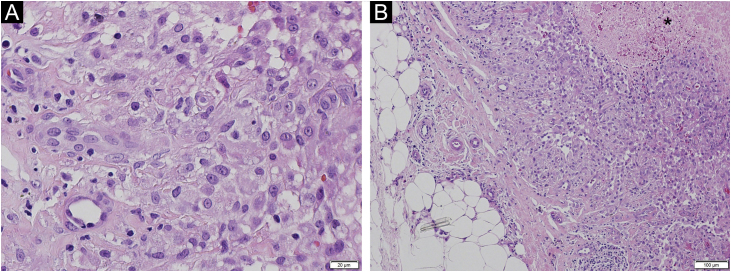
Histopathology of the nodular lesion (Hematoxylin & eosin): epithelioid cells with mild atypia and granulomatous arrangement (A, B). Dermo-hypodermic infiltrate and focal areas of necrosis (*) in the deep dermis (B).

ES is a rare subtype of soft tissue sarcoma with a high potential for local recurrence and metastases. It preferentially affects young male adults and about a quarter of cases are associated with previous trauma, such as the present one.^1^, ^2^, ^3^ It is subdivided into proximal type, with a bad prognosis, affecting the trunk, axilla, and perineum, usually presenting as infiltrating masses in the subcutaneous tissue, ^3^ and distal type (classic), usually occurring in the extremities as firm, painless, slow-growing nodules. Microscopically, the classic form is composed of epithelioid cells with abundant eosinophilic cytoplasm in a fascicular arrangement, surrounding a central necrotic area, producing a granulomatous pattern (Fig. 2).^1^, ^2^, ^3^ In the proximal variant, on the other hand, the cells have a rhabdoid morphology and increased atypia. There may be “signet-ring” vacuolation.^3^ ES is positive for both epithelial and mesenchymal markers. CD34 and epithelial membrane antigen are often positive.^3^ Typical negative markers include S100, desmin, CD68, and CD31.^2^ CD68 positivity is uncommon, but focal reaction in macrophages in ES associated with intense inflammatory infiltrate, as in the present case, has been reported.^2^ Such a finding is mostly seen in granulomatous processes such as rheumatoid nodules and granuloma annulare.^3^ A common characteristic of ES, present in more than 90% of cases, is the loss of expression of INI-1, the tumor suppressor gene expressed in normal nucleated cells.^3^, ^4^ A minority of cases of ES, as in the reported study, maintains normal INI-1 expression and may be related to a more aggressive biological behavior.^3^, ^4^

The differential diagnosis includes nodules due to infectious disease, rheumatoid nodules, fibrous histiocytoma, and other soft tissue sarcomas.^3^, ^4^ In the present case, the ascending trajectory of the lesions and their rapid evolution raised the hypothesis of lymphocutaneous sporotrichosis and cutaneous mycobacteriosis, which were ruled out by the cultures and histopathological analysis.

Surgery remains the mainstay of treatment in localized disease, with or without radiation, with disease-free surgical margins being the most important prognostic factor.^3^, ^4^, ^5^ Chemotherapy is the treatment of choice for advanced disease. Recently, tazemetostat, a histone methyltransferase inhibitor, was approved for the treatment of locally advanced or metastatic ES.^3^

## Financial support

None declared.

## Authors’ contributions

Vivian Spanemberg Macuglia: Approval of the final version of the manuscript; design and planning of the study; drafting and editing of the manuscript; intellectual participation in the propaedeutic and/or therapeutic conduct of the studied cases; critical review of the literature; critical review of the manuscript.

Juliano Peruzzo: Approval of the final version of the manuscript; design and planning of the study; drafting and editing of the manuscript; intellectual participation in the propaedeutic and/or therapeutic conduct of the studied cases; critical review of the literature; critical review of the manuscript.

Ariane Bastos Geller: Approval of the final version of the manuscript; design and planning of the study; drafting and editing of the manuscript; intellectual participation in the propaedeutic and/or therapeutic conduct of the studied cases; critical review of the literature; critical review of the manuscript.

Renan Rangel Bonamigo: Approval of the final version of the manuscript; design and planning of the study; drafting and editing of the manuscript; intellectual participation in the propaedeutic and/or therapeutic conduct of the studied cases; critical review of the literature; critical review of the manuscript.

## Conflicts of interest

None declared.
